# Mental health in Sierra Leone

**DOI:** 10.1192/bji.2019.17

**Published:** 2020-02

**Authors:** Dawn Harris, Tarik Endale, Unn Hege Lind, Stephen Sevalie, Abdulai Jawo Bah, Abdul Jalloh, Florence Baingana

**Affiliations:** 1 Mental Health Coordinator, King's Sierra Leone Partnership, King's Global Health Partnerships, King's Centre for Global Health and Health Partnerships, School of Population and Environmental Sciences, King's College London, Sierra Leone. Email: dawn.harris@kcl.ac.uk; 2 Mental Health Volunteer, King's Sierra Leone Partnership, King's Global Health Partnerships, King's Centre for Global Health and Health Partnerships, School of Population and Environmental Sciences, King's College London, Sierra Leone; 3 Mental Health Volunteer, King's Sierra Leone Partnership, King's Global Health Partnerships, King's Centre for Global Health and Health Partnerships, School of Population and Environmental Sciences, King's College London, Sierra Leone; 4 Commanding Officer, Joint Medical Unit, Republic of Sierra Leone Armed Forces, Sierra Leone; 5 Lecturer, Department of Pharmacology, College of Medicine and Allied Sciences, University of Sierra Leone, Sierra Leone; 6 Specalist Psychiatrist, Hospital Care Manager, Sierra Leone Psychiatric Teaching Hospital; and Lecturer, College of Medicine and Allied Health Sciences, University of Sierra Leone, Sierra Leone; 7 Independent Consultant, Sierra Leone

**Keywords:** Low- and middle-income countries, Sierra Leone, global mental health, mental health system, human rights

## Abstract

Sierra Leone is a West African country with a population of just over 7 million. Many Sierra Leoneans lived through the psychologically distressing events of the civil war (1991–2002), the 2014 Ebola outbreak and frequent floods. Traditionally, mental health services have been delivered at the oldest mental health hospital in sub-Saharan Africa, with no services available anywhere else in the country. Mental illness remains highly stigmatised. Recent advances include revision of the Mental Health Policy and Strategic Plan and the strengthening of mental health governance and district services. Many challenges lie ahead, with the crucial next steps including securing a national budget line for mental health, reviewing mental health legislation, systematising training of mental health specialists and prioritising the procurement of psychotropic medications. National and international commitment must be made to reduce the treatment gap and provide quality care for people with mental illness in Sierra Leone.

Sierra Leone is situated on the west coast of Africa, flanked by the Atlantic Ocean and bordered by Liberia and Guinea. The country has a young and rapidly growing population of 7 092 113 people (50.8% female, 40.8% <15 years, 41% urban, 51% literacy, 78% Muslim) (UNFPA, 2018). The country is divided into 16 districts, of which the Western Area Urban District (Freetown) is the most populous with 1.1 million people. Sierra Leone has 20 local dialects, with distinct cultural beliefs and practices. More than half (52.9%) of Sierra Leoneans live below the poverty line (UNFPA, 2018).

Many Sierra Leoneans have experienced traumatic events that are associated with negative mental health outcomes (Betancourt *et al*, 2015). These include a decade-long civil war which ended in 2002, the 2014 Ebola outbreak (Jalloh *et al*, 2018), and recurrent floods which in August 2017 caused a deadly mudslide that left an estimated 1141 dead or missing (The World Bank, 2017). Additionally, alcohol use is significantly higher than the regional average (WHO, 2014) and substance misuse, especially of marijuana and tramadol, are of increasing concern (Shackman & Price, 2013).

## Systematic issues facing mental health services

Sierra Leone is a low-income country with a gross domestic product (GDP) per capita of $653 and a Human Development Index ranking of 181 out of 188 countries. Approximately 21.7% of the GDP is spent on health, which amounts to $160 per capita. However, only 7.3% of that expenditure comes from the government, with the rest coming from out-of-pocket expenditures (33%), donor funding (46.9%) and non-governmental organisations (11.8%). There is no official budget line allocated to mental health (Sierra Leone Ministry of Health and Sanitation, 2017).

Government investment in human resources for health has been limited, with few doctors in training and poor retention. Almost 50% of the health workforce has not been absorbed onto the payroll (Sierra Leone Ministry of Health and Sanitation, 2017), resulting in informal payments or reliance on other sources of income (Wurie *et al*, 2016).

Sierra Leone has an estimated treatment gap of 98% for severe mental illness (Alemu *et al*, 2012). Historically, mental healthcare was delivered at ‘Kissy Lunatic Asylum’ which opened in 1820 and was the oldest asylum in sub-Saharan Africa (Akyeampong *et al*, 2015). Now referred to as the Sierra Leone Psychiatric Hospital (SLPH), it remains the country's only in-patient facility, treating up to 150 patients at a time. SLPH is highly stigmatised and suffers from chronic underfunding, limited human resources, a lack of basic facilities and frequent interruptions to medication supplies. This often results in restricted treatment options and the chaining of patients; however, this dire situation is now starting to improve with support from international partners. Sierra Leone continues to use the outdated Lunacy Act of 1902 which is highly discriminatory.

Stigma towards mental illness is a major issue and mental health literacy is extremely low. Mental illness is viewed as something that is brought upon oneself or as the result of supernatural activity (Alemu *et al*, 2012). A person exhibiting symptoms of mental illness may be called *crezman* or *ful ful* (pejorative terms indicating that the person is crazy or a fool). By the time patients present to formal mental health services it is usually after considerable investment at home or with one of the 37 000 traditional healers in the country (Alemu *et al*, 2012). A 2005 assessment suggested that up to 88% of people with mental disorders had seen a traditional healer before accessing psychiatric care (Jones *et al*, 2009). Although there is evidence of potential psychosocial benefits of traditional healing in Sierra Leone (Stark, 2006), these healing methods are varied and unregulated, leaving great potential for abuse such as prolonged chaining, beating, confinement and deprivation of food and water.

## Major developments and future challenges

### Mental health governance and leadership

In 2012 a national Mental Health Policy was launched, with an updated Mental Health Policy and Strategic Plan in the process of finalisation for 2019. Mental health is included in the Basic Package of Essential Health Services and various other national strategic policies and plans.

In December 2016 a Directorate for Noncommunicable Diseases and Mental Health was established within the Ministry of Health and Sanitation (MoHS) to coordinate services and oversee partner mental health activities. The Directorate is in the process of drafting new mental health legislation with the hope of protecting the human rights of people with mental illnesses. District focal persons are soon to be appointed to act as advocates for mental health in their local area.

### Mental health workforce

A cohort of twenty-one mental health nurses completed either certificate- or diploma-level courses in 2012/13 at the College of Medicine and Allied Health Sciences (COMAHS) in Freetown. Unfortunately, two nurses passed away, leaving nineteen in clinical practice. Two further mental health nurses have trained abroad. A second cohort of eight nurses started their training with COMAHS in January 2019. Additionally, eight healthcare professionals have completed an MSc in child and adolescent mental health (CAMH) in Nigeria, but only one nurse continues to practice solely as a CAMH specialist. The University of Makeni is currently running a diploma in counselling psychology.

Two Sierra Leonean psychiatrists returned to the country following completion of specialist training in 2016: one is the medical director at SLPH and the other is the commanding officer of military medical services. Additionally, there is a semi-retired psychiatrist who previously ran SLPH. There are no clinical psychologists or psychiatric social workers in public service.

The scarcity of educational opportunities and senior staff for training and supervision is a challenge. Sierra Leone lacks the capacity and infrastructure to conduct specialist training of additional psychiatrists. Furthermore, many of those who completed the certificate/diploma course are yet to be professionally recognised and remunerated as mental health nurses. A curriculum is in development for specialist community health officers (CHOs) but training has not begun. CHOs run most of the primary health services and are vital for planning the future mental health workforce.

### Service delivery

Mental health services remain highly centralised. There are no mental health in-patient beds outside of SLPH. At the secondary care level, mental health treatment is delivered by mental health nurses. They operate out-patient units at district hospitals, as well as a national CAMH service at Ola During Children's Hospital.

There is limited integration of mental health into services such as primary care, maternal and child health or HIV clinics. At the peripheral health unit level and below, there are no designated mental health workers. Training on the use of the World Health Organization's Mental Health Gap Action Programme (mhGAP) has been conducted for over 100 CHOs, however most are yet to receive any supervision or refresher training and the impact is still to be evaluated.

### Health management information systems and research

There has been a dearth of mental health research in Sierra Leone, with the last systematic epidemiological study dating back to a 2002 MoHS survey (Alemu *et al*, 2012). Subsequent mental health research has largely focused on the civil war and Ebola.

Currently, MoHS routine data collection has been relegated to a single box labelled ‘mental disorder,’ which is rarely completed. This lack of disaggregated mental health data limits usefulness for service planning and policy action. Partners have worked with mental health nurses to strengthen monitoring and evaluation systems through the collection of routine clinic data. Although there are concerns about accuracy, the data provides some insight into service utilisation and diagnosis. From 2015 to 2017, epilepsy accounted for almost half of the diagnoses, with psychosis appearing almost twice as frequently as depression or alcohol and substance misuse.

### Health financing and medical products

At present, there is no specific mental health budget line at the level of the MoHS or the District Health Management Teams. Psychotropics are included on the list of essential medications but have rarely been procured by the MoHS; most psychotropics in the country are donations. The lack of mental health funding and a severe shortage of psychotropics at the district level are major barriers for accessing treatment. Limited options at all levels hinder the provision of quality services and the retention and motivation of mental health workers.

## Conclusions

There is a long way to go before inhumane practices are put to an end and minimum standards of treatment are reached, but the establishment of a Directorate for Noncommunicable Diseases and Mental Health demonstrates a growing commitment to the cause. Although mental health is included in the Minimum Package of Essential Health Services and the Health Policy and Strategic Plan, this has not translated into adequate investment towards the strengthening of the mental health system. Efforts to improve research and health information systems are crucial to future planning. It will also take significant investment in human resources, with training of additional psychiatrists and the development of a local Mental Health BSc for nurses and CHOs. Further efforts to decentralise mental healthcare are also key. The launch of the national Strategic Plan and Policy will be vital to guide the improvement of services. Programmes to increase mental health literacy among the population are also needed. This will require the involvement of stakeholders both within and outside of the formal health system, such as traditional/faith healers. Together, these steps will help pave the way for the future delivery of high-quality, universal mental healthcare in Sierra Leone.
